# Salvage Interstitial Brachytherapy for Isolated Local Recurrence of Cervical and Endometrial Cancer: A Retrospective Analysis Stratified by Type of Pelvic Irradiation History

**DOI:** 10.3390/cancers18020252

**Published:** 2026-01-14

**Authors:** Den Fujioka, Takashi Saito, Taisuke Sumiya, Keiichiro Baba, Motohiro Murakami, Haruko Numajiri, Hiroya Itagaki, Ayumi Shikama, Yuri Tenjimbayashi, Azusa Akiyama, Sari Nakao, Masashi Mizumoto, Kei Nakai, Toyomi Satoh, Hideyuki Sakurai

**Affiliations:** 1 Department of Radiation Oncology, Faculty of Medicine, University of Tsukuba, Tsukuba 305-8575, Japan; dfujioka@pmrc.tsukuba.ac.jp (D.F.); sumiya@pmrc.tsukuba.ac.jp (T.S.); baba@pmrc.tsukuba.ac.jp (K.B.); murakami@pmrc.tsukuba.ac.jp (M.M.); haruko@pmrc.tsukuba.ac.jp (H.N.); mizumoto@pmrc.tsukuba.ac.jp (M.M.); knakai@pmrc.tsukuba.ac.jp (K.N.); hsakurai@pmrc.tsukuba.ac.jp (H.S.); 2 Department of Gynecologic Oncology, Faculty of Medicine, University of Tsukuba, Tsukuba 305-8575, Japan; itagaki-h@md.tsukuba.ac.jp (H.I.); ashikama@md.tsukuba.ac.jp (A.S.); yuri_tenj@md.tsukuba.ac.jp (Y.T.); aakiyama@md.tsukuba.ac.jp (A.A.); s.nakao@md.tsukuba.ac.jp (S.N.); toyomi-s@md.tsukuba.ac.jp (T.S.)

**Keywords:** cervical cancer, endometrial cancer, recurrent, re-irradiation, interstitial brachytherapy

## Abstract

**Background/Objectives**: This study evaluated the efficacy and safety of salvage interstitial brachytherapy (S-ISBT) for isolated local recurrence (ILR) of cervical and endometrial carcinoma, stratifying patients by pelvic irradiation history (PIH). **Methods**: Patients with ILR treated with S-ISBT were retrospectively reviewed and categorized by initial treatment: Group A (surgery alone); Group B (surgery + postoperative radiotherapy (RT)); and Group C (definitive RT). Overall survival (OS), progression-free survival (PFS), local control (LC) rates, and the cumulative incidence functions (CIFs) for Grade ≥ 3 late adverse events (AEs) were estimated. Multivariate analysis identified prognostic factors. **Results**: The study included 70 patients (A: 28, B: 17, C: 25) with a median follow-up of 33.4 months. The 3-year OS, PFS, LC, and CIFs for Grade ≥ 3 late AEs for Groups A, B, and C were 80.8%, 66.7%, and 30.4% (*p* < 0.001); 56.4%, 41.5%, and 11.6% (*p* < 0.001); 89.1%, 61.4%, and 43.0% (*p* = 0.002); and 26.4%, 13.3%, and 32.0% (*p* = 0.40), respectively. Multivariate analysis suggested the type of PIH, disease-free interval, and tumor volume as independent prognostic factors. While no significant differences were observed between Groups A and B (OS: HR = 0.47, *p* = 0.19; PFS: HR = 0.60, *p* = 0.28), Group C exhibited a significantly higher risk than Group B (OS: HR = 3.08, *p* = 0.018; PFS: HR = 3.41, *p* = 0.004). **Conclusions**: S-ISBT could be considered for patients with prior postoperative RT, whose outcomes are significantly better than those with prior definitive RT.

## Simple Summary

Pelvic recurrence of cervical and endometrial cancer is challenging to treat, especially in patients with a pelvic irradiation history (PIH). This study analyzed 70 patients treated with salvage interstitial brachytherapy (S-ISBT) for isolated local recurrence. Patients were grouped based on their initial treatment: (Group A) surgery alone, (Group B) surgery + postoperative radiotherapy (RT), or (Group C) definitive RT. Group B demonstrated superior overall survival (OS) and progression-free survival (PFS) compared to Group C. These findings suggest that the type of PIH is a key prognostic factor, and S-ISBT is a particularly viable salvage option for patients with prior postoperative RT.

## 1. Introduction

Locoregional recurrence rates after initial radical treatment range from 6–11% for cervical cancer and 2–20% for endometrial cancer [1,2,3]. Treatment for isolated local recurrence (ILR) of cervical and endometrial cancer varies depending on the site of recurrence and pelvic irradiation history (PIH) [4,5]. Cases of ILR of these malignancies without PIH can undergo curative treatment with multimodal approaches such as surgery, external-beam radiotherapy (EBRT), and brachytherapy, which provide favorable disease control. However, for ILR of cervical and endometrial cancer with PIH, re-irradiation is challenging due to the tolerable radiation dose limits for surrounding organs at risk (OARs). In such cases, the indication for local treatment has to be considered carefully, with salvage treatment options including surgical resection (pelvic exenteration or total hysterectomy), brachytherapy, and stereotactic body radiotherapy (SBRT). Surgical resection can be effective for central recurrences localized to the vagina or cervix but is generally limited to cases without pelvic wall involvement. Moreover, the procedure is associated with high rates of severe adverse events (AEs) (34.7–65.3%) and mortality (0.98–6%) [6,7,8]. For unresectable ILR with PIH, curative treatment options are severely limited. In these instances, precision radiotherapy (RT) techniques, including brachytherapy and SBRT, are the primary modalities for a potential cure. Salvage interstitial brachytherapy (S-ISBT) is particularly effective for re-irradiation in gynecologic cancers, as it allows for precise and efficient dose delivery to the tumor [9].

The disease-free interval (DFI), recurrent tumor volume, and the site of relapse are well-known favorable prognostic factors for ILR of gynecologic cancer [9,10,11], whereas the impact of the type of PIH is less clear. Many studies do not distinguish prior irradiation used postoperatively from definitive RT. This distinction is critical because definitive RT generally uses a higher radiation dose, which may make recurrent tumors more difficult to treat due to greater radio-resistance. Indeed, recurrent tumors often exhibit increased radio-resistance compared to primary tumors, driven by biological mechanisms such as vascular, stromal, and immunological changes induced within the tumor microenvironment [12], along with the selection of radio-resistant cell clones during initial therapy.

This study was motivated by our clinical impression that outcomes of S-ISBT vary substantially depending on whether the prior radiation was postoperative RT or definitive RT. We hypothesized that this distinction is essential because definitive RT typically involves higher prior doses, which often results in a clinical observation of poorer treatment response in the recurrent tumor and stricter physical constraints for re-irradiation compared to postoperative RT. Therefore, the aim of this study is to evaluate the clinical outcomes of patients treated with S-ISBT for ILR of cervical and endometrial cancer and to validate whether the type of PIH (none, postoperative RT, or definitive RT) is a significant prognostic factor to guide clinical decision-making.

## 2. Materials and Methods

### 2.1. Patient Population

**Figure 1:**
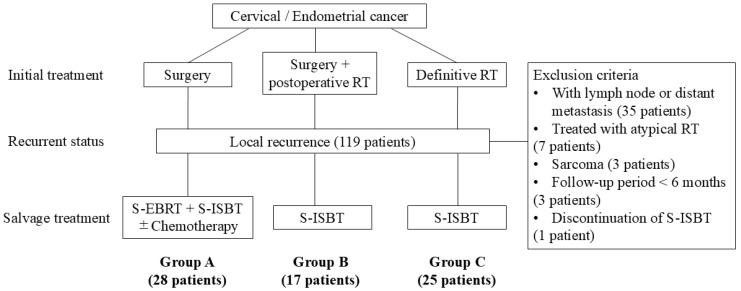
Initial treatment and classification of patient groups. RT, radiotherapy; S-EBRT, salvage external-beam radiotherapy; S-ISBT, salvage interstitial brachytherapy.

This retrospective study was approved by the Institutional Review Board of the Tsukuba Clinical Research & Development Organization. Data were extracted from medical records and the treatment planning system for cases treated from September 2010 to September 2022. Patients who underwent S-ISBT for ILR of cervical or endometrial cancer after initial radical treatment were included. ILR was defined as a pelvic local recurrence without radiological evidence of lymph-node or distant metastasis at the time of recurrence. ILR was diagnosed based on a combination of physical examination and imaging findings. Radiological evidence of local recurrence was defined as the appearance of a new lesion or progressive enlargement of an existing mass exhibiting high signal intensity on T2-weighted MRI and/or intense fluorodeoxyglucose uptake on PET-CT. The exclusion criteria were lymph node metastasis or distant metastasis at the time of recurrence, surgery after neoadjuvant RT, sarcoma diagnosis, a follow-up period < 6 months after treatment in survivors, or discontinuation of S-ISBT due to withdrawal of consent for reasons unrelated to AEs. Patients were classified into three groups based on initial treatment (Figure 1): surgery (Group A), surgery and postoperative RT (Group B), and definitive RT (Group C). Typically, the previous radiation dose was 50 Gy in 25 fractions (fr) of pelvic EBRT for Group B, and 50 Gy of pelvic EBRT and brachytherapy of 24 Gy in 4 fr for Group C. However, detailed dose-volume histogram parameters from this prior RT, such as target volume D90 and D2cc for OARs, were largely unavailable for review. Recurrence sites were classified as central or pelvic wall according to established criteria [13]. For cases with a PIH, the ILR lesion was considered to overlap if it was even partly in the previous radiation field, and not to overlap if the lesion was entirely outside this field. DFI was defined as the time from the end of initial treatment to the date of histologic or definitive radiographic confirmation of ILR.

### 2.2. Salvage Treatment Methods

Different treatment strategies for ILR of cervical or endometrial cancer were used for each group: patients without PIH (Group A) received salvage EBRT (S-EBRT) followed by S-ISBT in 3–5 fr. Conversely, for patients with PIH (Groups B and C), S-EBRT was omitted to mitigate the risk of exceeding normal tissue tolerance limits due to prior irradiation, and thus, these cases received S-ISBT alone in 6–7 fr. Throughout the study period (2010–2022), all S-ISBT procedures were performed using a uniform CT-based image-guided protocol. To ensure consistent and accurate target definition, the clinical target volume (CTV) was defined as the recurrent tumor plus a 5–10 mm margin, based on findings from gynecologic examination, CT and/or T2-weighted MRI. In Group A, S-EBRT was delivered to the pelvis by three-dimensional conformal RT using a four-field box technique with 10 MV X-rays, which included CTV and elective irradiation of the pelvic lymph node region. The timing of central shielding and concurrent chemotherapy was determined by clinicians. In accordance with the methodology described in [14], brachytherapy was performed in all groups using an iridium-192 (192Ir) remote after-loading system (MicroSelectron HDR-TM; Nucletron, Veenendaal, The Netherlands). The applicators used included a vaginal cylinder and a Martinez Universal Perineal Interstitial Template secured to the perineum. Metal or plastic needles were inserted through these applicators under spinal/epidural or intravenous anesthesia. The insertion depth was adjusted to maximize CTV coverage while sparing OARs. Planning CT images were acquired with a slice thickness of 2.5–5 mm, and contouring was performed on these images using the latest MRI as a reference. The prescribed dose was 6 Gy per fr, with the dosimetric goal of covering at least 90% of the CTV with the prescribed dose (D90 ≥ 6 Gy). Irradiation was performed twice daily with an interval of at least 6 h. Dosimetric constraints for OARs were adjusted according to the type of PIH. For Group A, constraints followed standard definitive RT protocols, as exemplified by a recent Japanese prospective multi-institutional study [15]. In contrast, for Groups B and C (re-irradiation), dose prescriptions were optimized on a case-by-case basis by clinicians to achieve an optimal balance between sufficient tumor coverage and cumulative normal tissue tolerance.

### 2.3. Dose-Volume Analysis

Oncentra Brachy (Nucletron, Veenendaal, The Netherlands) was utilized for treatment planning and to acquire information on CTV D90, rectal D2cc, and bladder D2cc. For combining the treatment doses of S-EBRT and S-ISBT, the biologically effective dose (BED) was first calculated using a linear-quadratic model:

(1) BED = *nd* (1 + *d*/(α/β)),

where *n* is the number of fr and *d* is the dose per fr. The α/β ratio was assumed to be 10 for the tumor and 3 for the bladder and rectum. The BED was then converted to the equivalent dose in 2 Gy fr (EQD2). The dose from the central shield was excluded from the total dose calculations for CTV D90 and OARs.

### 2.4. Follow-Up and Statistical Analysis

Examinations were conducted every 1–3 months for the first 3 years after S-ISBT, and every 6 months thereafter. CT or MRI was reviewed every 3–6 months. The time to event for all endpoints was defined as the period from the start of salvage treatment to the first event or the date of the last follow-up. Local recurrence after salvage treatment was defined as radiologically suspicious local disease progression or pathological malignant findings of the treated gross tumor volume (GTV) in the uterus, vagina, or vulva. For this retrospective study, all clinical data and late AEs were collected by reviewing paper and/or electronic medical records. We primarily used detailed progress notes and imaging reports from scheduled follow-up visits. Late AEs were defined as those occurring > 3 months after S-ISBT and were graded according to CTCAE v5.0. Baseline patient characteristics were compared among the three groups. The Wilcoxon rank-sum and Kruskal–Wallis tests were used for continuous variables, while Fisher’s exact test was used for categorical variables. Overall survival (OS) and progression-free survival (PFS) were estimated using the Kaplan–Meier method and compared among Groups A, B, and C by the log-rank test. For local control (LC) and grade ≥ 3 late AEs, a competing-risk approach was used, with death considered a competing-risk. The cumulative incidence functions (CIFs) were estimated with the Aalen–Johansen estimator and compared among Groups A, B, and C using Gray’s test. For display, LC calculated as 1—CIFs of local failure, while toxicity was shown as CIFs. To identify prognostic factors for OS and PFS, a multivariate Cox proportional hazards model was used for all patients. Selection of variables for the model was based on univariate analysis, with *p* < 0.2 used as the criterion for inclusion. The primary variable of interest, the treatment group, was included in the final model regardless of its univariate *p*-value. For this variable, Group B was used as the reference category because it generally represents an intermediate prior radiation exposure between Groups A and C, enabling clinically meaningful comparisons. Initial International Federation of Gynecology and Obstetrics (FIGO) stage and pathology were not considered as candidate variables for this analysis due to their strong correlation with the treatment group definitions. The proportional hazards assumption was assessed using Schoenfeld residuals (global and covariate-level tests). All statistical analyses were performed using R software (v.4.4.3; R Foundation for Statistical Computing, Vienna, Austria).

## 3. Results

### 3.1. Patient and Tumor Characteristics

As shown in the study flow diagram (Figure 1 in Section 2), the final cohort comprised 70 patients stratified into three groups based on their initial treatment: Group A (*n* = 28), Group B (*n* = 17), and Group C (*n* = 25). The patient characteristics are shown in Table 1. Some data were unavailable, including histology for one patient in Group C and FIGO stage for two patients in Group B, due to missing records from referring institutions. Additionally, tumor volume was unavailable for four patients in Group A (no recent MRI) and one patient in Group C (non-measurable disease on MRI). As expected from the treatment-based stratification, there were significant differences among the groups in primary disease site, histology, and initial FIGO stage (*p* < 0.001). In contrast, other factors, including age, performance status (PS), DFI, and tumor volume were comparable across the groups. For patients with PIH (Groups B and C), the recurrent GTV overlapped with the prior treatment field in 39 of 42 cases (93%). A small number of patients underwent preparatory procedures before S-ISBT, including colostomy (*n* = 3) and spacer insertion (*n* = 3).

### 3.2. Salvage Treatment Details

The salvage treatment details for each group are shown in Table 2. In Group A (*n* = 28), S-EBRT combined with S-ISBT was performed in all cases. The S-EBRT dose was 50 Gy in 25 fr (*n* = 25), 50.4 Gy in 28 fr (*n* = 2), and 30 Gy in 15 fr (*n* = 1). A central shield was used in 25 cases. The S-ISBT dose was 30 Gy in 5 fr (*n* = 20), 24 Gy in 4 fr (*n* = 6), and 18 Gy in 3 fr (*n* = 2). Concurrent chemotherapy of cisplatin alone (*n* = 15) or cisplatin + paclitaxel (*n* = 1) was administered to 16 patients in Group A. In Groups B (*n* = 17) and C (*n* = 25), all patients received S-ISBT alone, with treatment delivered in 6 fr (*n* = 8) or 7 fr (*n* = 34). Concurrent chemotherapy was not used in these groups. A median of 13 interstitial needles was used across all patients (range: 3–24). In Groups A, B and C, respectively, the median total dose to the CTV D90 EQD2 (α/β = 10) was 73.1, 58.3, and 57.3 Gy; and the median D2cc EQD2 (α/β = 3) for OARs was 70.5, 34.0, and 45.8 Gy for the bladder, and 62.6, 34.5, and 40.0 Gy for the rectum.

### 3.3. Survival and Local Control

**Figure 2:**
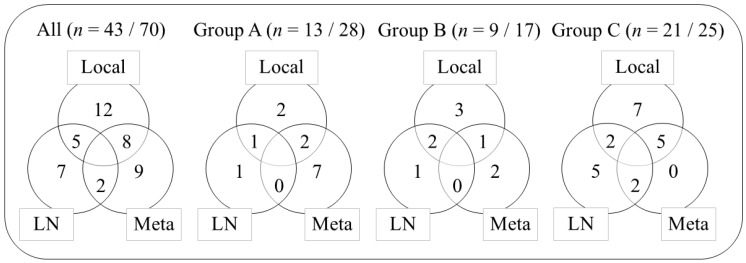
Number of relapses after salvage treatment. LN, lymph node; Meta, metastasis.

**Figure 3:**
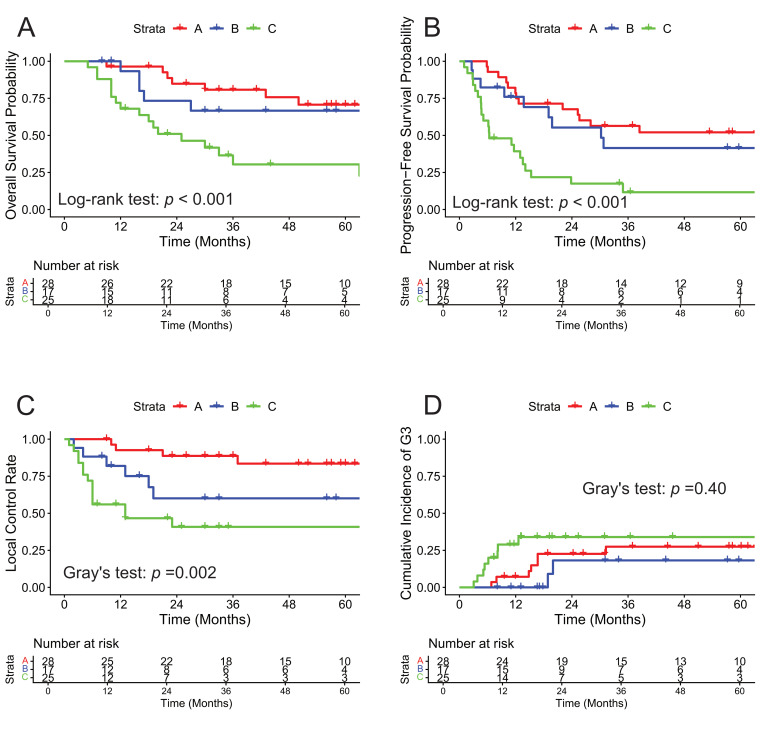
Survival outcomes and late toxicities. (**A**) Overall survival and (**B**) progression-free survival (Kaplan–Meier; log-rank test). (**C**) Local control (1—CIFs of local failure) and (**D**) CIFs for Grade ≥ 3 late AEs (the Aalen–Johansen estimator; death as a competing risk; Gray’s test). AEs, adverse events; CIFs, cumulative incidence functions.

The median follow-up period after salvage treatment was 33.4 (5.3–128.6) months for all cases and 57.1 (8.1–128.6) months for survivors. During follow-up, 43 patients (61%) had recurrence. The patterns of failure included local recurrence (*n* = 25), lymph node recurrence (*n* = 14: pelvic (*n* = 3), extra-pelvic (*n* = 6), both (*n* = 5)) and distant organ metastasis (*n* = 19); and some patients presented with multiple sites of recurrence (Figure 2). A total of 31 patients died, including 30 (97%) cause-specific deaths. The survival and LC rates for each group are shown in Figure 3A–C. In Groups A, B, and C, respectively, the 3-year OS rates were 80.8% (95% CI: 67.0–97.5%), 66.7% (95% CI: 46.6–95.3%), and 30.4% (95% CI: 15.8–58.5%) (*p* < 0.001); the 3-year PFS rates were 56.4% (95% CI: 40.5–78.4%), 41.5% (95% CI: 22.7–75.8%), and 11.6% (95% CI: 3.5–38.3%) (*p* < 0.001); and the 3-year LC rates (1—CIFs) were 89.1% (95% CI: 77.3–100%), 61.4% (95% CI: 35.9–87.0%), and 43.0% (95% CI: 22.4–63.6%) (*p* = 0.002). Analyses restricted to cervical cancer (*n* = 48) showed similar trends (Figure S1). The 3-year outcomes for Groups A, B, and C were OS of 64.8%, 61.5%, and 30.4% (*p* = 0.078); PFS of 45.7%, 30.8%, and 11.6% (*p* = 0.033); LC of 88.9%, 53.8%, and 43.0% (*p* = 0.01).

### 3.4. Analysis of Prognostic Factors for Overall Survival and Progression-Free Survival

The results of univariate and multivariate analyses for OS and PFS are shown in Table 3. Because tumor volume was missing in five patients, the multivariate model including tumor volume was fitted in complete cases (*n* = 65). In multivariate analyses, treatment group, DFI, and tumor volume were independently associated with both OS and PFS. Specifically, a history of definitive RT (Group C vs. B) and a larger tumor volume were associated with worse outcomes for OS (HR = 3.08, *p* = 0.018; HR = 1.04, *p* < 0.001) and PFS (HR = 3.41, *p* = 0.004; HR = 1.02, *p* = 0.003). Conversely, a longer DFI was a significant protective factor for OS (HR = 0.98, *p* = 0.003) and PFS (HR = 0.99, *p* = 0.007). The proportional hazards assumption was not violated (Schoenfeld residuals; global test: OS, *p* = 0.61; PFS, *p* = 0.40). A supplementary model excluding tumor volume was fitted in the full cohort (*n* = 70), yielding consistent results for treatment group (Table S1). Only a history of definitive RT (Group C vs. B) associated with worse outcomes for OS (HR = 2.79, *p* = 0.031) and PFS (HR = 2.73, *p* = 0.013).

### 3.5. Late Adverse Events

Grade 3 or higher late AEs occurred in 18 patients (25.7%). The 3-year CIFs for Grade ≥ 3 late AEs were 26.4% (95% CI: 9.1–43.8%) in Group A, 13.3% (95% CI: 0–31.4%) in Group B, and 32.0% (95% CI: 13.2–50.8%) in Group C (Figure 3D); no significant statistical differences were observed among the groups (*p* = 0.40). Analyses restricted to cervical cancer (*n* = 48) showed similar trends, with 3-year CIFs for Grade ≥ 3 late AEs of 37.1%, 15.4%, and 32.0% in Groups A, B, and C, respectively (*p* = 0.42) (Figure S1). A total of 23 distinct Grade 3 or higher AEs were recorded in the 18 patients (Table 4). The most frequent AE was vaginal fistula (*n* = 13). For the management of these fistulas, surgical interventions such as urinary diversion or stoma construction were performed as necessary. One Grade 5 vaginal fistula occurred in a patient in Group C. This fatal case involved local recurrence directly invading the vaginal wall and bladder, causing vaginal fistula and subsequent sepsis. Others included urinary tract obstruction (*n* = 2), ileus (*n* = 2), rectal hemorrhage (*n* = 2), hematuria (*n* = 2), vaginal inflammation (*n* = 1), and edema limbs (*n* = 1).

## 4. Discussion

In this study, we investigated the effectiveness of S-ISBT for ILR of cervical and endometrial carcinoma by categorizing cases based on the type of initial treatment. A distinctive feature of the study is the analysis of cases with PIH divided into postoperative and definitive RT. The postoperative RT cases had better outcomes than the definitive RT cases, despite the groups having no significant differences in background factors unrelated to initial treatment, such as age, PS, local recurrent site, DFI, and tumor volume. These findings suggest the need to consider both the use of previous irradiation and the radiation dose administered in evaluating potential outcomes of ILR.

Several retrospective reports have evaluated the treatment outcomes of RT for ILR of cervical and endometrial cancer. As expected, outcomes for ILR without PIH were most favorable, showing an OS of 43–79.4%, PFS of 45–62.7%, and LC of 69–100% at 3–8 years [16,17,18,19,20]. In this study, the 3-year OS, PFS, and LC in cases without PIH were 80.8%, 56.4%, and 89.1%, respectively, which are consistent with previous results. Although several studies have reported on RT for cases with PIH, few have calculated treatment outcomes separately for postoperative and definitive RT. Table 5 summarizes the findings of previous reports that categorized PIH into these two groups [21,22,23]. These reports indicate a trend that cases with a history of postoperative RT had significantly better outcomes than those with definitive RT. In a similar analysis, Yoshida et al. found that S-ISBT for ILR of uterine cancer with a history of postoperative irradiation had better LC rates compared to cases with a history of definitive irradiation [21]. However, the study had a small sample size and did not examine OS and PFS, whereas we were able to include these endpoints in multivariate analysis. Our results suggested that cases with a history of postoperative RT had significantly better OS and PFS compared to those with definitive RT, although these findings should be interpreted as associative rather than confirmatory due to the small number of events (Table 3).

The difference in outcomes between postoperative (Group B) and definitive (Group C) RT cases may be attributed to both clinical and biological factors. First, Group B tended to have more favorable clinical backgrounds. Specifically, Group B exhibited a relatively smaller tumor volume and more prolonged DFI compared to Groups A and C, although these differences did not reach statistical significance (Table 1). Importantly, however, our multivariate analysis suggested that the type of PIH was associated with prognosis even after adjusting for tumor volume and DFI. This finding may underscore that the prognostic impact of prior radiation type is not merely a reflection of these clinical backgrounds but is an inherent factor influencing outcomes. Second, biological differences in radio-resistance likely played a role. Radio-resistance is a major cause of poor prognosis and treatment failure. Recent advances have begun to clarify the complex mechanisms underlying this resistance caused by epigenetic modifications [24,25,26]. Recurrences following high-dose definitive RT are presumed to be highly radioresistant; in contrast, the doses typically used in postoperative RT are relatively lower than those in definitive RT, suggesting that recurrent tumors in Group B may retain higher radiosensitivity. While the biological mechanisms of radio-resistance remain speculative and require further validation, these clinical findings highlight the potential significance of the type of PIH.

Interestingly, the 3-year CIFs for Grade ≥ 3 late AEs were lowest in Group B (13.3%), followed by Group A (26.4%), and highest in Group C (32.0%) (Figure 3D). This difference is likely influenced by the specific clinical characteristics of each group and the intensity of prior RT. Recurrences in Group B were typically smaller and more centrally located than those in the other two groups (Table 1). These clinical features likely facilitated easier and more precise applicator and needle placement, which could have contributed to reducing doses to the bladder and the rectum (Table 2). Although late toxicity remains a significant concern across all groups, S-ISBT appears to be a particularly viable and well-tolerated option for patients with a history of postoperative RT. In contrast, Group C exhibited a high rate of late AEs and local recurrence with frequent fistula formation. Clinically, it was often difficult to distinguish whether these fistulas resulted from tumor invasion or complications directly attributable to S-ISBT. In fact, as shown in Table 4, several cases of vaginal fistula occurred in patients who also experienced local recurrence. Furthermore, because RT is closely linked to immune and inflammatory mechanisms [27], re-irradiation in a previously high-dose field might theoretically trigger immune exhaustion, further increasing the risk of severe AEs in Group C. However, such biological hypotheses remain speculative in the absence of biomarker data. Such intensive prior exposure may lead to profound tissue fibrosis and a reduced tolerance for re-irradiation. The high incidence of complications in Group C likely reflects both the frequent local recurrence and the burden of higher prior RT doses compared to Group B. However, the presence of long-term survivors in Group C suggests that S-ISBT remains a viable curative option for carefully selected patients, even if it represents a clinically challenging approach.

Comparison of the effectiveness of S-ISBT with other salvage treatments is essential. For patients with resectable central recurrences after PIH, pelvic exenteration can offer excellent long-term survival, with 5-year OS rates as high as 41.2–72% [28,29]. However, this radical surgery is associated with high rates of severe AEs and mortality. Furthermore, a direct comparison between surgical outcomes and our study results is limited by inherent selection bias as our cohort consisted of unresectable recurrences. Importantly, our findings suggest that S-ISBT serves as a curative option for patients who are unsuitable for salvage surgery. For patients who are not surgical candidates, systemic chemotherapy has been the standard of care. Historically, chemotherapy alone has offered limited efficacy, with a median OS of approximately 18 months [30,31]. While our S-ISBT outcomes showed a longer median OS (22 months even for Group C), a straightforward comparison is not appropriate due to the heterogeneity of historical chemotherapy cohorts, which often include patients with distant metastases at baseline. Recently, advances in immune checkpoint inhibitors (ICIs) have significantly improved the prognosis for recurrent gynecologic cancers [32,33,34]. In our study, distant metastasis was a common pattern of recurrence following S-ISBT, suggesting that LC alone may be insufficient. However, due to concerns regarding treatment-related toxicity in the re-irradiation setting, concurrent chemotherapy was not administered to Groups B and C. Future strategies should focus on integrating S-ISBT with modern systemic therapy to address both local and systemic control, thereby optimizing long-term outcomes for patients with ILR.

The limitations of this study include its single-center, retrospective design, and potential referral bias as patients referred to our specialized center for S-ISBT may not fully represent the general population of patients with ILR. Although cervical and endometrial cancers were analyzed together due to the rarity of ILR, the strong correlation between histology and PIH groups introduced inherent confounding bias. Furthermore, the limited number of events may have led to model instability; thus, our multivariate findings should be interpreted as associative rather than confirmatory. Additionally, the lack of detailed prior dosimetric data may have compromised the accuracy of our evaluation of late toxicities, as our assessment was restricted to prescribed doses. Moreover, the increasing clinical use of ICIs during the long study period (2010–2022) may also have influenced the outcomes. Therefore, it is necessary to standardize S-ISBT protocols across institutions and consider conducting prospective studies to evaluate its efficacy more accurately. Future research should focus on integrating S-ISBT with systemic therapies to improve outcomes, particularly for high-risk patients with a history of definitive RT.

## 5. Conclusions

In this study, we evaluated the efficacy and safety of S-ISBT for locally recurrent cervical and endometrial cancer, stratified by the type of PIH. Importantly, patients with a history of postoperative RT (Group B) showed favorable OS and PFS rates compared to those who received definitive RT (Group C). These findings suggest the effectiveness of S-ISBT as a re-treatment option and indicate that the type of prior irradiation could be a critical determinant of prognosis in the salvage setting. Further prospective multicenter studies are warranted to validate these results and optimize patient selection.

## Figures and Tables

**Table 1:** Patient characteristics.

Characteristics		Group A (*n* = 28)	Group B (*n* = 17)	Group C (*n* = 25)	*p*-Value
Age (years)	Median (range)	63 (33–83)	58 (41–78)	63 (36–87)	0.736
PS (ECOG)	0	19	10	19	0.613
	1	8	7	5	
	2	1	0	1	
Primary disease site	Cervix	10	13	25	<0.001
	Endometrium	18	4	0	
Histology	SCC	6	10	20	<0.001
	Adenocarcinoma	20	7	3	
	Adenosquamous	0	0	1	
	Others	2	0	0	
	Unknown	0	0	1	
FIGO 2008 stage	I	24	6	2	<0.001
	II	1	7	11	
	III	3	2	10	
	IV	0	0	2	
	Unknown	0	2	0	
Prior radiation field	Overlap	0	15	24	0.556 *^1^
	Separate	28	2	1	
Prior radiation modality	EBRT	0	11	0	<0.001 *^1^
	ICBT	0	3	0	
	EBRT + ICBT	0	3	25	
Prior radiation dose (Gy, EQD2)	Median (range)	None	50.0 (32.0–66.0)	72.0 (56.0–93.3)	<0.001 *^2^
Local recurrent site	Central	19	13	17	0.799
	Pelvic wall	9	4	8	
DFI (months)	Median (range)	18.8 (0.93–165.8)	20.3 (6.0–132.2)	11.7 (1.1–166.3)	0.199
Tumor volume (cc) *^3^	Median (range)	16.2 (1.4–180.7)	6.7 (1.0–72.6)	16.7 (3.5–88.7)	0.155

DFI, disease-free interval; EBRT, external-beam radiotherapy; ECOG, Eastern Cooperative Oncology Group; EQD2, Equivalent dose in 2 Gy fractions; FIGO, International Federation of Gynecology and Obstetrics; ICBT, intracavitary brachytherapy; PS, performance status; SCC, squamous cell carcinoma. *^1^ Comparison between Group B and C by Fisher’s exact test. *^2^ Comparison between Group B and C by the Wilcoxon rank-sum test. *^3^ Tumor volume data were missing for 5 patients.

**Table 2:** Details of salvage treatment in each group.

Characteristics		Group A (*n* = 28)	Group B (*n* = 17)	Group C (*n* = 25)
S-EBRT dose	50 Gy/25 fr	25	0	0
	50.4 Gy/28 fr	2	0	0
	30 Gy/15 fr	1	0	0
	None	0	17	25
Central shield at S-EBRT	Yes	25	0	0
	No	3	17	25
S-ISBT dose	18 Gy/3 fr	2	0	0
	24 Gy/4 fr	6	0	0
	30 Gy/5 fr	20	0	0
	36 Gy/6 fr	0	2	6
	42 Gy/7 fr	0	15	19
Concurrent chemotherapy	Cisplatin	15	0	0
	Cisplatin + Paclitaxel	1	0	0
	None	12	17	25
CTV D90 Total dose * (Gy, EQD2)	Median (range)	73.1 (62.8–102.4)	58.3 (44.1–68.6)	57.3 (48.5–65.5)
OARs D2cc Total dose * (Gy, EQD2)	Bladder Median (range)	70.5 (27.7–92.8)	34.0 (6.2–76.4)	45.8 (3.0–61.2)
	Rectum Median (range)	62.6 (25.4–77.6)	34.5 (14.6–64.5)	40.0 (4.0–75.8)

CTV, clinical target volume; D2cc, minimum dose delivered to the most irradiated 2 cc volume of an organ; D90, minimum dose delivered to 90% of the target volume; EQD2, equivalent dose in 2 Gy fractions; fr, fractions; OARs, organs at risk; S-EBRT, salvage external-beam radiotherapy; S-ISBT, salvage interstitial brachytherapy. * Total dose of only salvage treatment.

**Table 3:** Relationships of variables with overall survival and progression-free survival.

Variables	Category	Overall Survival				Progression-Free Survival			
		Univariate (31/70 Events)		Multivariate (31/65 Events) *		Univariate (43/70 Events)		Multivariate (41/65 Events) *	
		HR (95% CI)	*p*-Value	HR (95% CI)	*p*-Value	HR (95% CI)	*p*-Value	HR (95% CI)	*p*-Value
Age (years)		0.98 (0.96–1.01)	0.26	—	—	0.99 (0.96–1.01)	0.24	—	—
PS (ECOG)	0	1 (ref)	—	—	—	1 (ref)	—	—	—
	1, 2	0.87 (0.39–1.95)	0.73	—	—	0.93 (0.46–1.76)	0.82	—	—
Group	B	1 (ref)	—	1 (ref)	—	1 (ref)	—	1 (ref)	—
	A	0.59 (0.20–1.75)	0.34	0.47 (0.15–1.47)	0.19	0.73 (0.31–1.71)	0.47	0.60 (0.23–1.53)	0.28
	C	2.86 (1.13–7.24)	0.027	3.08 (1.21–7.85)	0.018	2.71 (1.23–5.98)	0.01	3.41 (1.48–7.88)	0.004
Local recurrent site	Central	1 (ref)	—	—	—	1 (ref)	—	—	—
	Pelvic wall	1.55 (0.75–3.19)	0.24	—	—	1.04 (0.53–2.03)	0.91	—	—
DFI (month)		0.99 (0.98–1.00)	0.15	0.98 (0.97–0.99)	0.003	0.99 (0.98–1.00)	0.17	0.99 (0.98–1.00)	0.007
Tumor volume (cc) *		1.01 (1.00–1.02)	0.005	1.04 (1.02–1.05)	<0.001	1.01 (1.00–1.02)	0.11	1.02 (1.01–1.03)	0.003

CI, confidence interval; DFI, disease-free interval; ECOG, Eastern Cooperative Oncology Group; HR, hazard ratio; PS, performance status; ref, reference. * Univariate (tumor volume) and all multivariate analyses used complete cases (*n* = 65); 5 patients had missing tumor volume.

**Table 4:** Cases with late adverse events of Grade 3 or higher.

No.	Group	S-ISBT to AEs Interval (Months)	Late AEs	Grade	S-EBRT Dose (Gy)	S-ISBT Dose (Gy)	Local Recurrence	Complication	Antithrombotic Drugs
1	C	3.0	Vaginal fistula	3	0	42	Yes	No	No
2	C	3.8	Vaginal fistula	4	0	42	Yes	No	No
3	C	5.1	Vaginal fistula	5	0	42	Yes	HT/DM/Stroke	Yes
			Urinary tract obstruction	3					
4	C	5.4	Vaginal fistula	3	0	42	Yes	No	No
			Rectal hemorrhage	3					
5	C	6.2	Vaginal fistula	3	0	42	Yes	No	No
6	A	6.8	Ileus	3	40	24	No	No	No
7	A	7.9	Ileus	3	40	28	No	DVT	Yes
8	C	8.1	Vaginal fistula	3	0	42	Yes	No	No
9	C	8.2	Vaginal fistula	3	0	42	No	No	No
10	C	12.6	Vaginal fistula	3	0	42	No	HT	No
			Hematuria	3					
11	A	14.8	Vaginal fistula	3	40	30	Yes	No	No
12	A	15.3	Vaginal fistula	4	40	24	Yes	HT	No
13	A	16.7	Vaginal fistula	3	30	30	No	No	No
14	A	16.7	Vaginal inflammation	3	30	30	No	HT	No
15	B	18.9	Urinary tract obstruction	3	0	42	Yes	HT	No
16	B	20.0	Vaginal fistula	3	0	42	Yes	No	No
17	A	31.3	Vaginal fistula	3	40	30	Yes	No	No
			Hematuria	3					
			Rectal hemorrhage	3					
18	A	95.1	Edema limbs	3	50	30	No	HT	No

AEs, adverse events; DM, diabetes mellitus; DVT, deep vein thrombosis; HT, hypertension; S-EBRT, salvage external-beam radiotherapy; S-ISBT, salvage interstitial brachytherapy.

**Table 5:** Published reports of re-irradiation for recurrent cervical/endometrial cancer stratified by the type of pelvic irradiation history.

Author	*n*	Prior RT	Re-Irradiation Method	Median DFI (Months) (Range)	Median CTV D90 (Gy, EQD2) (Range)	OS (Year)	PFS (Year)	LC (Year)
Yoshida et al. [21]	8	Postoperative	ISBT	N/A	N/A	N/A	N/A	75% (3)
	13	Definitive						46% (3)
Liu et al. [22]	16	Postoperative	ISBT	<12: 56.3% >12: 43.7%	52.5 (49.2–55.8)	N/A	N/A	CR: 37.5% PR: 43.8%
Ling et al. [23]	22	Postoperative	ICBT/ISBT ± EBRT	26.6	64.5 (49.6–75.8)	68.1% (3)	40.8% (3)	65.8% (3)
Current study	17	Postoperative	ISBT	20.3 (6.0–132.2)	58.3 (44.1–68.6)	66.7% (3)	41.5% (3)	61.4% (3)
	25	Definitive		11.7 (1.1–166.3)	57.3 (48.5–65.5)	30.4% (3)	11.6% (3)	43.0% (3)

CR, complete response; CTV, clinical target volume; D90, minimum dose delivered to 90% of the target volume; DFI, disease-free interval; EBRT, external-beam radiotherapy; EQD2, equivalent dose in 2 Gy fractions; ICBT, intracavitary brachytherapy; ISBT, interstitial brachytherapy; LC, local control; N/A, not applicable; OS, overall survival; PFS, progression-free survival; PR, partial response; RT, radiotherapy.

## Data Availability

The datasets used and/or analyzed during the current study are available from the corresponding author on reasonable request.

## Supplementary Materials

The following supporting information can be downloaded at: https://www.mdpi.com/article/10.3390/cancers18020252/s1, Table S1. Relationships of variables with overall survival and progression-free survival, excluding tumor volume. Figure S1. Survival outcomes and late toxicities in the cervical cancer subgroup. (A) Overall survival and (B) progression-free survival (Kaplan–Meier; log-rank test). (C) Local control (1—CIFs of local failure) and (D) CIFs for Grade ≥ 3 late AEs (the Aalen–Johansen estimator; death as a competing risk; Gray’s test). AEs, adverse events; CIFs, cumulative incidence functions.

## Abbreviations

The following abbreviations are used in this manuscript:

AEs	Adverse events
BED	Biologically effective dose
CI	Confidence interval
CIFs	Cumulative incidence functions
CR	Complete response
CT	Computed tomography
CTCAE	Common terminology criteria for adverse events
CTV	Clinical target volume
D2cc	Minimum dose delivered to the most irradiated 2 cc volume of an organ
D90	Minimum dose delivered to 90% of the target volume
DFI	Disease-free interval
DM	Diabetes mellitus
DVT	Deep vein thrombosis
EBRT	External-beam radiotherapy
ECOG	Eastern Cooperative Oncology Group
EQD2	Equivalent dose in 2 Gy fractions
FIGO	International Federation of Gynecology and Obstetrics
fr	Fractions
GTV	Gross tumor volume
HDR	High-dose-rate
HR	Hazard ratio
HT	Hypertension
ICBT	Intracavitary brachytherapy
ICIs	Immune checkpoint inhibitors
ILR	Isolated local recurrence
LC	Local control
LN	Lymph node
Meta	Metastasis
MRI	Magnetic resonance imaging
N/A	Not applicable
OARs	Organs at risk
OS	Overall survival
PET	Positron emission tomography
PFS	Progression-free survival
PIH	Pelvic irradiation history
PR	Partial response
PS	Performance status
ref	reference
RT	Radiotherapy
SBRT	Stereotactic body radiotherapy
SCC	Squamous cell carcinoma
S-EBRT	Salvage external-beam radiotherapy
S-ISBT	Salvage interstitial brachytherapy
