# Supplementary material for: Salvage Interstitial Brachytherapy for Isolated Local Recurrence of Cervical and Endometrial Cancer: A Retrospective Analysis Stratified by Type of Pelvic Irradiation History
Source: Cancers (Basel). 2026 Jan 14;18(2):252. doi: 10.3390/cancers18020252 (PMC12838614; doi:10.3390/cancers18020252)
Supplement: Supplementary file 1 [file cancers-18-00252-s001.zip › cancers-4040850-supplementary.pdf]

Table S1. Relationships of variables with overall survival and progression-free survival, excluding tumor volume.

Variables	Category	Overall Survival Multivariate (31/ 70 events) HR (95%CI)	Overall Survival Multivariate (31/ 70 events) *p*-value	Progression-free Survival Multivariate (43/ 70 events) HR (95%CI)	Progression-free Survival Multivariate (43/ 70 events) *p*-value
Group	B	1 (ref)	—	1 (ref)	—
	A	0.56 (0.19–1.68)	0.304	0.68 (0.29–1.61)	0.384
	C	2.79 (1.10–7.08)	0.031	2.73 (1.24–6.04)	0.013
DFI (month)		0.99 (0.98–1.00)	0.139	0.99 (0.98–1.00)	0.137

CI, confidence interval; DFI, disease-free interval; HR, hazard ratio; ref, reference.

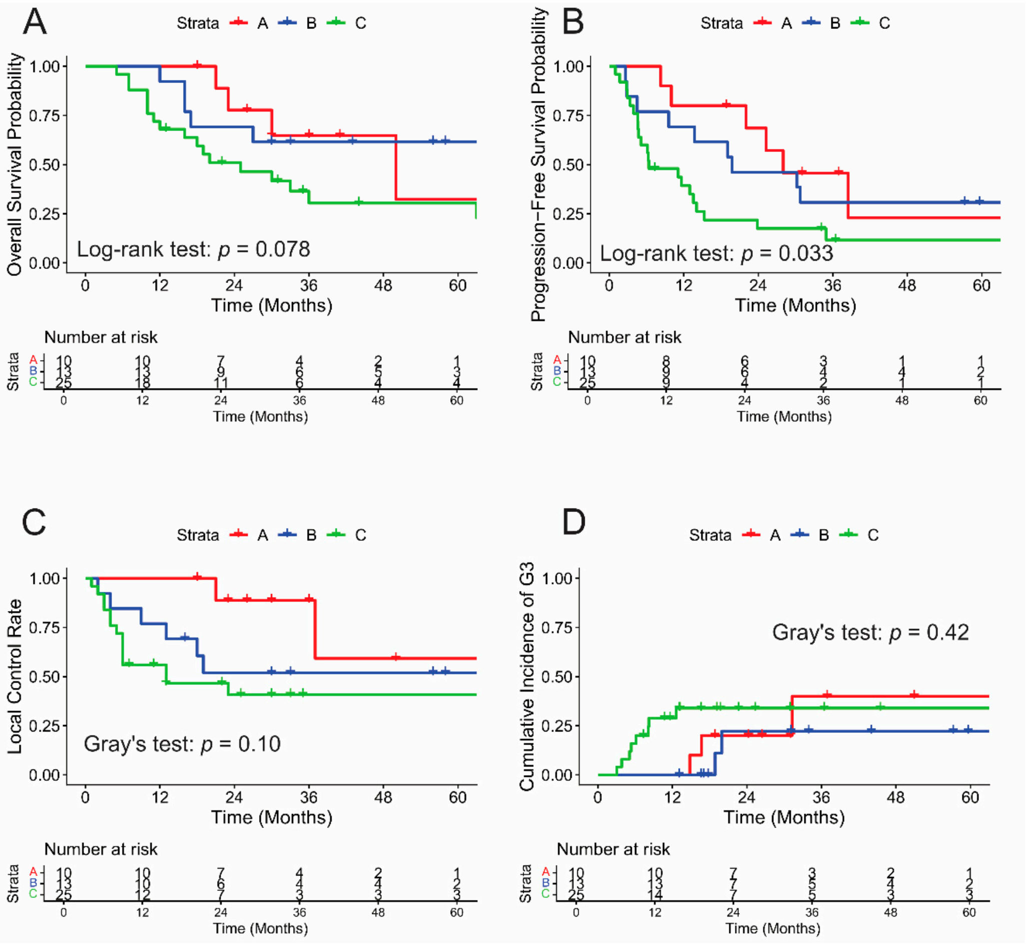

Figure S1. Survival outcomes and late toxicities in the cervical cancer subgroup. (A) Overall survival and (B) progression-free survival (Kaplan–Meier; log-rank test). (C) Local control (1—CIFs of local failure) and (D) CIFs for Grade ≥ 3 late AEs (the Aalen–Johansen estimator; death as a competing risk; Gray’s test). AEs, adverse events; CIFs, cumulative incidence functions.
